# Machine learning to predict futile recanalization of large vessel occlusion before and after endovascular thrombectomy

**DOI:** 10.3389/fneur.2022.909403

**Published:** 2022-08-19

**Authors:** Xinping Lin, Xiaohan Zheng, Juan Zhang, Xiaoli Cui, Daizu Zou, Zheng Zhao, Xiding Pan, Qiong Jie, Yuezhang Wu, Runze Qiu, Junshan Zhou, Nihong Chen, Li Tang, Chun Ge, Jianjun Zou

**Affiliations:** ^1^School of Basic Medicine and Clinical Pharmacy, China Pharmaceutical University, Nanjing, China; ^2^Department of Pharmacy Nanjing First Hospital, Nanjing Medical University, Nanjing, China; ^3^Department of Neurology, Nanjing Yuhua Hospital, Yuhua Branch of Nanjing First Hospital, Nanjing Medical University, Nanjing, China; ^4^Department of Pharmacy, Nanjing First Hospital, China Pharmaceutical University, Nanjing, China; ^5^Department of Neurology, Nanjing First Hospital, Nanjing Medical University, Nanjing, China; ^6^Department of Pharmacy, Yixing Cancer Hospital, Yixing, China

**Keywords:** futile recanalization, machine learning, large vessel occlusion, endovascular thrombectomy, predictive model

## Abstract

**Background and purpose:**

Futile recanalization occurs when the endovascular thrombectomy (EVT) is a technical success but fails to achieve a favorable outcome. This study aimed to use machine learning (ML) algorithms to develop a pre-EVT model and a post-EVT model to predict the risk of futile recanalization and to provide meaningful insights to assess the prognostic factors associated with futile recanalization.

**Methods:**

Consecutive acute ischemic stroke patients with large vessel occlusion (LVO) undergoing EVT at the National Advanced Stroke Center of Nanjing First Hospital (China) between April 2017 and May 2021 were analyzed. The baseline characteristics and peri-interventional characteristics were assessed using four ML algorithms. The predictive performance was evaluated by the area under curve (AUC) of receiver operating characteristic and calibration curve. In addition, the SHapley Additive exPlanations (SHAP) approach and partial dependence plot were introduced to understand the relative importance and the influence of a single feature.

**Results:**

A total of 312 patients were included in this study. Of the four ML models that include baseline characteristics, the “Early” XGBoost had a better performance {AUC, 0.790 [95% confidence intervals (CI), 0.677–0.903]; Brier, 0.191}. Subsequent inclusion of peri-interventional characteristics into the “Early” XGBoost showed that the “Late” XGBoost performed better [AUC, 0.910 (95% CI, 0.837–0.984); Brier, 0.123]. NIHSS after 24 h, age, groin to recanalization, and the number of passages were the critical prognostic factors associated with futile recanalization, and the SHAP approach shows that NIHSS after 24 h ranks first in relative importance.

**Conclusions:**

The “Early” XGBoost and the “Late” XGBoost allowed us to predict futile recanalization before and after EVT accurately. Our study suggests that including peri-interventional characteristics may lead to superior predictive performance compared to a model based on baseline characteristics only. In addition, NIHSS after 24 h was the most important prognostic factor for futile recanalization.

## Introduction

Endovascular thrombectomy (EVT) is standard-of-care in patients with large vessel occlusion (LVO) stroke of the anterior circulation according to the latest international guidelines (1). Its benefit and safety have been repeatedly underlined in a series of randomized clinical trials (RCTs) (2). The therapeutic target of EVT is to achieve recanalization to improve long-term functional outcomes. However, futile recanalization considered a poor long-term function outcome despite adequate vessel recanalization, remains a common phenomenon. Previous studies showed that the incidence of futile recanalization among LVO patients ranged from 47 to 67%, and these futile recanalization cases may occur due to poor microvascular compromise, poor collateral circulation, technology difference, and cerebral blood flow regulation (3–7). Patients with futile recanalization undergoing EVT may suffer reperfusion injury and consume resources and time, so early prediction of futile recanalization is critical.

Many predictors associated with futile recanalization in LVO patients undergoing EVT have been reported. Baseline clinical characteristics such as advanced age, female gender, and severe neurological deficits, have been reported to be correlated with futile recanalization (3, 8). Neuroimaging characteristics such as baseline Alberta Stroke Program Early Computed Tomography Score (ASPECTS), poor collateral circulation, and final infarction volume, have also been suggested as important factors (3–5). There are also peri-interventional characteristics such as general anesthesia and delayed puncture to reperfusion (9). However, all the predictors are based on traditional statistical algorithms and even if all those predictors are taken into account, it should be emphasized that they are not efficient in perfectly predicting futile recanalization in LVO patients. Therefore, to improve individual stroke care, it is crucial to establish a reliable and data-driven model that integrates information from various sources (clinical, neuroimaging, peri-interventional characteristics) to accurately predict futile recanalization in LVO patients and differentiate between them based on whether they will or will not benefit from EVT. Unfortunately, no reliable models are designed to predict futile recanalization in stroke patients subjected to EVT.

Machine learning (ML) can analyze kinds of characteristics and leverage the integrated predictive value of these characteristics. Moreover, the ML approach can detect non-linear relationships in clinical data and uncover new patterns from existing information. Indeed, ML algorithms have already been proven to help predict functional outcomes after endovascular treatment in ischemic stroke patients (10), classify stroke mechanisms (11), and detect early infarction from non-contrast-enhanced CT (12). Notably, these sophisticated computer algorithms have gained significant interest in the widespread use of electronic health record systems and the accessibility of data from patients.

Here, we aimed to evaluate the prognostic factors associated with futile recanalization using ML algorithms and develop a pre-EVT model (the “Early” model) and a post-EVT model (the “Late” model) to effectively predict the risk of futile recanalization, and more importantly, to improve stroke emergency care and provide patients' relatives with reliable information about the prognosis.

## Materials and methods

### Study design and population

Consecutive acute ischemic stroke (AIS) patients receiving EVT at the National Advanced Stroke Center of Nanjing First Hospital (China) between April 2017 and May 2021 were included in the study. All patients had a clinically confirmed diagnosis of AIS with LVO of the anterior circulation and underwent EVT according to the standard of care using stent-retrievers and/or aspiration catheters at the Department of Neurology at Nanjing First Hospital. Patients were selected if they fulfilled the following criteria: (1) age 18 years or older; (2) LVO in the anterior circulation, including the internal carotid artery (ICA), M1/M2 segment of the middle cerebral artery (MCA); (3) successful recanalization [defined as modified Thrombolysis In Cerebral Infarction (mTICI) scale grades 2b or 3]; (4) premorbid modified Rankin Scale (mRS) score ≤ 2; (5) known National Institutes of Health Stroke Scale (NIHSS), ASPECTS, and mRS score at 90 days; (6) time from onset to puncture ≤ 6 or 6–24 h with evidence of perfusion mismatch. Patients who missed more than one data were to be excluded. The flowchart is summarized in Supplementary Figure S1. We dichotomized eligible patients with mTICI ≥ 2b into two groups utilizing the 90-day mRS score, which included the futile recanalization (90-day mRS of 3–6) and meaningful recanalization (90-day mRS of 0–2). The 90-day mRS scores were assessed *via* telephone-based interview or outpatient visit 3 months after onset. The scientific use of the data was approved by the Ethics Committee of Nanjing First Hospital (document number: KY20130424-01) and all patients provided written informed consent.

### Data collection and definitions

Demographics and clinical characteristics were recorded on admission. We also collected data on treatment information and complication. More details of the definition can be found in the Supplementary methods.

### Feature selection and model development

To assess the accumulative predictive power of clinical, neuroimaging, and peri-interventional characteristics, we built two ML models to predict futile recanalization risk: The first “Early” model was based on baseline clinical and neuroimaging characteristics at admission. The second “Late” model was developed *via* all variables from the “Early” model + peri-interventional characteristics. For model development, the original dataset was randomly stratified into training and test sets per 8:2, which meant that the proportion of patients with futile recanalization in the two sets was consistent with the original dataset. Then, the least absolute shrinkage and selection operator (LASSO) regression, a sparse method, was performed to select the important features in the training set. Furthermore, all features determined by LASSO were introduced into the four ML models to assess futile recanalization risk. This included logistic regression with L2 regularization (LR with L2), random forest classifier (RFC), support vector machine (SVM), and extreme gradient boosting (XGBoost). To avoid overfitting, we utilized a grid search algorithm with 10-fold cross-validation to fine-tune the optimal hyperparameters (Supplementary Tables S1A, S1B) in the training set. A separate test set was used to assess the models' generalization performance. In addition, all continuous variables were standardized using *Z*-score normalization. The algorithms involved above were performed in Python 3.7 using Scikit-learn version 0.24.1 and XGboost version 1.2.1 libraries.

### Model evaluation and interpretation

We focused on discrimination and calibration to evaluate the performance of the “Early” and the “Late” model. The discrimination was mirrored using the area under the receiver operating characteristic curve (AUC), and the Delong test (13) was applied to describe the statistical difference of AUC. In addition, the following metrics: sensitivity, specificity, positive predictive value, negative predictive value, and accuracy, were also calculated accordingly. Calibration ability was assessed by the Brier score, which calculated the difference between real-world and model-predicted index outcomes. A lower score indicated better calibration. Furthermore, the incremental benefit of ML model calibration was compared using the null model Brier score (14).

To better understand the predictive process of the ML model, we applied the model-agnostic interpretability techniques, including the feature importance and partial dependence plot (PDP) (15). The feature importance was performed by the Shapley Additive exPlanations (SHAP) algorithm (16). This sorting process is based on the mean of absolute SHAP values for all individuals. PDP was introduced to help understand how a single feature influences futile recanalization. These interpretability techniques were implemented in Python using SHAP version 0.39.0 and PDPbox version 0.2.0.

### Statistical analyses

All analyses were conducted using SPSS version 25.0. Initially, missing values were supplemented per the multiple imputation method. Then continuous variables were tested for normality. Notably, premorbid mRS and NIHSS scores on admission were regarded as continuous variables in all analyses for increasing the model's efficiency. All variables were shown with descriptive statistics. Univariate analyses were performed using the Student *t*-test or Mann-Whitney *U*-test for continuous variables, when appropriate. And Fisher's exact test or the χ2 test were applied for categorical variables.

## Results

### General condition

Out of 569 patients, 312 patients were eligible. Overall, 179 developed futile recanalization. As shown in Table 1A, those who developed futile recanalization were more likely to be older {median age: 75 [interquartile range (IQR), 67–81] vs. 66 [IQR, 60–76]} and suffered from more severe strokes [median NIHSS score on admission: 16 (IQR, 12–20) vs. 11 (IQR, 8–16)] compared with patients with meaningful recanalization. Furthermore, as seen in Table 1B participants with futile recanalization spent more time in the procedure of groin to recanalization [median of 72 min (IQR, 53–95) vs. 56 min (IQR, 43–75)] and had greater postprocedural NIHSS after 24 h [median of 16 (IQR, 12–21) vs. 5 (IQR, 3–10)]. Furthermore, all characteristics were well-balanced between the training and test sets (Supplementary Tables S2A, S2B).

**Table 1A:** Demographics and clinical characteristics.

	**Total** **(*n* = 312)**	**Meaningful recanalization** **(*n* = 133, 42.6%)**	**Futile recanalization** **(*n* = 179, 57.4%)**	***p*-value**
Baseline characteristics				
Age, years, median (IQR)	72.00 (63.25–79.00)	66 (60–76)	75 (67–81)	<0.001
Male sex, *n* (%)	186 (59.6)	89 (66.9)	97 (54.2)	0.023
BMI, kg/m^2^, median (IQR)	23.88 (21.48–26.67)	24.22 (22.04–26.70)	23.66 (21.22–26.12)	0.167
Education, years, *n* (%)				0.280
0–6	174 (55.8)	67 (50.4)	107 (59.8)	
6–9	67 (21.5)	29 (21.8)	38 (21.2)	
9–12	42 (13.5)	22 (16.5)	20 (11.2)	
>12	29 (9.3)	15 (11.3)	14 (7.8)	
Premorbid mRS (IQR)	0 (0–0)	0 (0–0)	0 (0–0)	<0.001
NIHSS on admission, median (IQR)	14 (11–18)	11 (8–16)	16 (12–20)	<0.001
Baseline SBP, mmHg, mean (SD)	138.02 (23.24)	137.36 (23.36)	138.51 (23.21)	0.665
Baseline DBP, mmHg, mean (SD)	84.02 (15.01)	83.09 (14.44)	84.70 (15.42)	0.348
Risk factors of vessels				
Hypertension, *n* (%)	236 (75.6)	95 (71.4)	141 (78.8)	0.135
Diabetes mellitus, *n* (%)	101 (32.4)	39 (29.3)	62 (34.6)	0.321
Dyslipidemia, *n* (%)	76 (24.4)	35 (26.3)	41 (22.9)	0.488
Coronary artery disease, *n* (%)	62 (19.9)	25 (18.8)	37 (20.7)	0.682
Atrial fibrillation, *n* (%)	99 (31.7)	37 (27.8)	62 (34.6)	0.201
Previous ischemic stroke/TIA, *n* (%)	67 (21.5)	24 (18)	43 (24)	0.204
Previous hemorrhagic stroke, *n* (%)	4 (1.3)	0 (0)	4 (2.2)	0.139
Smoking, *n* (%)				0.002
Never smoker	191 (61.2)	67 (50.4)	124 (69.3)	
Former smoker	23 (7.4)	11 (8.3)	12 (6.7)	
Current smoker	98 (31.4)	55 (41.4)	43 (24)	
Drinking, *n* (%)				<0.001
Never drinker	224 (71.8)	85 (63.9)	139 (77.7)	
Former drinker	15 (4.8)	3 (2.3)	12 (6.7)	
Current drinker	73 (23.4)	45 (33.8)	28 (15.6)	
Radiological baseline characteristics				
ASPECTS on admission, median (IQR)	5 (4–7)	5 (4–7)	5 (4–7)	0.083
Cause of stroke, *n* (%)				
LAA	121 (38.8)	58 (43.6)	63 (35.2)	0.131
CE	158 (50.6)	58 (43.6)	100 (55.9)	0.032
SAO	4 (1.3)	3 (2.3)	1 (0.6)	0.316
SOC	8 (2.6)	7 (5.3)	1 (0.6)	0.012
SUC	21 (6.7)	7 (5.3)	14 (7.8)	0.372
Vascular occlusion site, *n* (%)				
ICA	97 (31.1)	39 (29.3)	58 (32.4)	0.561
MCA M1	194 (62.2)	84 (63.2)	110 (61.5)	0.759
MCA M2	21 (6.7)	10 (7.5)	11 (6.1)	0.632
Side of occlusion, *n* (%)				
Left	147 (47.1)	64 (48.1)	83 (46.4)	0.759
Right	151 (48.4)	63 (47.4)	88 (49.2)	0.754
Both side	14 (4.5)	6 (4.5)	8 (4.5)	0.986
Medication use history				
Previous antiplatelet, *n* (%)	43 (13.8)	17 (12.8)	26 (14.5)	0.659
Previous anticoagulation, *n* (%)	26 (8.3)	10 (7.5)	16 (8.9)	0.654
Previous statin, *n* (%)	29 (9.3)	14 (10.5)	15 (8.4)	0.518

IQR, interquartile range; SD, standard deviation; BMI, body mass index; mRS, modified Ranking Scale; NIHSS, National Institutes of Health Stroke Scale; SBP, systolic blood pressure; DBP, diastolic blood pressure; TIA, transient ischemic attacks; ASPECTS, Alberta Stroke Program Early CT Score; LAA, large artery atherosclerosis; CE, cardioembolism; SAO, small artery occlusion; SOC, stroke of other determined cause; SUC, stroke of undetermined cause; ICA, internal carotid artery; MCA, middle cerebral artery.

**Table 1B:** Treatment information and complication.

	**Total** **(*n* = 312)**	**Meaningful recanalization** **(*n* = 133, 42.6%)**	**Futile recanalization** **(*n* = 179, 57.4%)**	***p*-value**
Treatment information
Intravenous thrombolysis, *n* (%)	138 (44.2)	63 (47.4)	75 (41.9)	0.336
Number of passages, *n* (%)	2 (1–3)	1 (1–2)	2 (1–3)	<0.001
Onset to emergency, min, median (IQR)	150.00 (60.50–287.50)	150 (60–295)	145 (65–278)	0.894
Onset to image, min, median (IQR)	194.00 (120.75–331.50)	215 (120–347)	190 (123–320)	0.420
Onset to groin, min, median (IQR)	259.00 (185.00–406.75)	270 (185–420)	251 (185–380)	0.444
Onset to recanalization, min, median (IQR)	342.50 (249.25–474.00)	340 (240–510)	344 (259–460)	0.926
Groin to recanalization, min, median (IQR)	64.50 (49.00–89.00)	56 (43–75)	72 (53–95)	<0.001
Later than 6 h from onset to puncture, *n*(%)	93 (29.8)	45 (33.8)	48 (26.8)	0.180
Later than 8 h from onset to puncture, *n*(%)	55 (17.6)	25 (18.8)	30 (16.8)	0.640
mTICI score, *n* (%)				0.387
2b	126 (40.4)	50 (37.6)	76 (42.5)	
3	186 (59.6)	83 (62.4)	103 (57.5)	
NIHSS after 24 h	12 (6–17)	5 (3–10)	16 (12–21)	<0.001
Post-treatment blood pressure variability
SBP
SD, median (IQR)	11.54 (7.42–16.91)	11.06 (7.54–16.36)	11.89 (7.33–17.24)	0.351
CV, median (IQR)	0.09 (0.06–0.13)	0.09 (0.06–0.12)	0.09 (0.06–0.13)	0.591
DBP
SD, median (IQR)	8.48 (5.63–11.21)	8.73 (5.59–11.61)	8.17 (5.79–11.15)	0.809
CV, median (IQR)	0.11 (0.08–0.15)	0.11 (0.08–0.15)	0.11 (0.08–0.14)	0.932
Complications
Brain edema, *n* (%)	14 (4.5)	0 (0)	14 (7.8)	0.001
END, *n* (%)	39 (12.5)	5 (3.8)	34 (19)	<0.001
sICH, *n* (%)	9 (2.9)	0 (0)	9 (5)	0.012

IQR, interquartile range; mTICI, modified Thrombolysis in Cerebral Infarction; SBP, systolic blood pressure; DBP, diastolic blood pressure; SD, standard deviation; CV, coefficient of variation; NIHSS, National Institutes of Health Stroke Scale; END, early neurological deterioration; sICH, symptomatic intracranial hemorrhage.

### “Early” models

**Figure 1:**
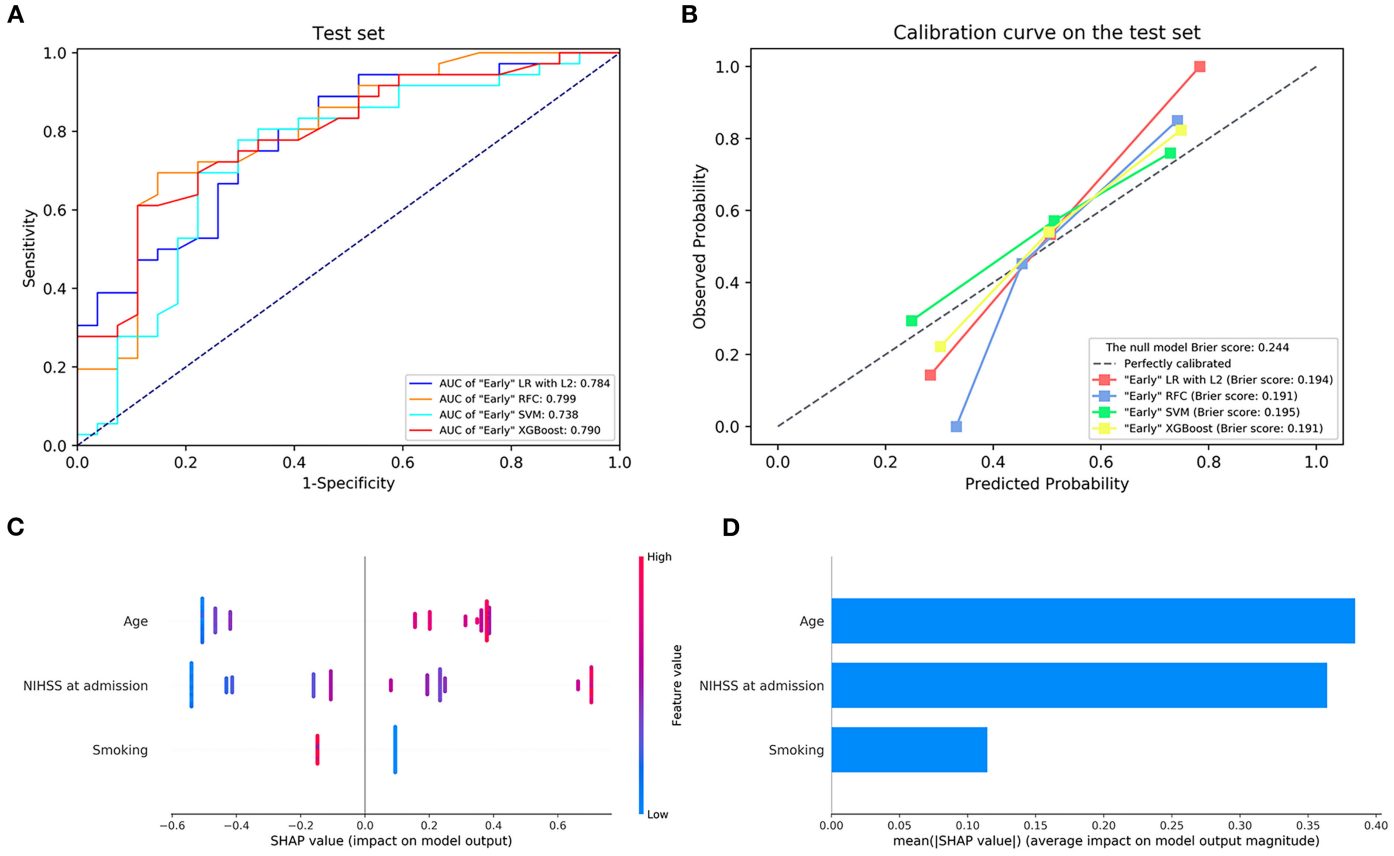
**(A)** The receiver operating characteristic curve and **(B)** the calibration curve of the “Early” machine learning models on the testing set. **(C,D)** Feature importance ranking based on Shapley Additive exPlanations (SHAP) values in “Early” XGBoost. AUC, area under the curve; LR with L2, logistic regression with L2 regularization; SVM, support vector machine; RFC, random forest classifier; XGBoost, extreme gradient boost. NIHSS, National Institutes of Health Stroke Scale.

The details of the model's performance on the training set are shown in Supplementary Figure S2 and Supplementary Table S3A. In terms of discriminatory ability, there were no significant differences in AUCs in all the “Early” ML models, AUCs ranged from 0.738 to 0.799 on the test set. Nevertheless, when considering the calibration, the overall performance of the XGBoost model was better than other ML models revealed by a smaller Brier score. For the sake of simplicity, we only considered the XGBoost as a prediction model. A summary of the results is given in Figure 1, Table 2A, and Supplementary Table S4A.

**Table 2A:** Scores of each “Early” model on the test set.

**Model**	**AUC (95% CI)**	**Sensitivity**	**Specificity**	**PPV**	**NPV**	**Accuracy**	**Brier score**
LR with L2	0.784 (0.671–0.898)	0.806	0.593	0.725	0.696	0.714	0.194
RFC	0.799 (0.686–0.913)	0.722	0.704	0.765	0.655	0.714	0.191
SVM	0.738 (0.606–0.870)	0.750	0.704	0.771	0.679	0.730	0.195
XGBoost	0.790 (0.677–0.903)	0.556	0.889	0.870	0.600	0.698	0.191

AUC, the area under the receiver operating characteristic curve; CI, confidence intervals; PPV: positive predictive value; NPV, negative predictive value; LR with L2, logistic regression with L2 regularization; RFC, random forest classifier; SVM, support vector machine; XGBoost, extreme gradient boosting.

Next, the visual interpretation of the “Early” model (XGBoost) was provided. Sorted by the mean absolute SHAP value, the rank of feature importance in descending order was as follows: age, NIHSS score on admission, and smoking. A dot in Figure 1C represents an individual. Red indicates the larger distribution of SHAP values, while blue indicates smaller. The high SHAP values of age and NIHSS reveal positive contributions to futile recanalization, whereas smoking was negative. Additionally, PDP shows the impact of each feature on the predicted risk (Supplementary Figure S3). For the age feature, although the impact appears to be a little fluctuating, on average, the impact increases drastically with age from 63 to 71 years. And it remains stable at other ages. For the NIHSS score between 6 and 22, on average, the higher the NIHSS, the larger the risk. And then, the impact remains constant after NIHSS of 22.

### “Late” models

**Figure 2:**
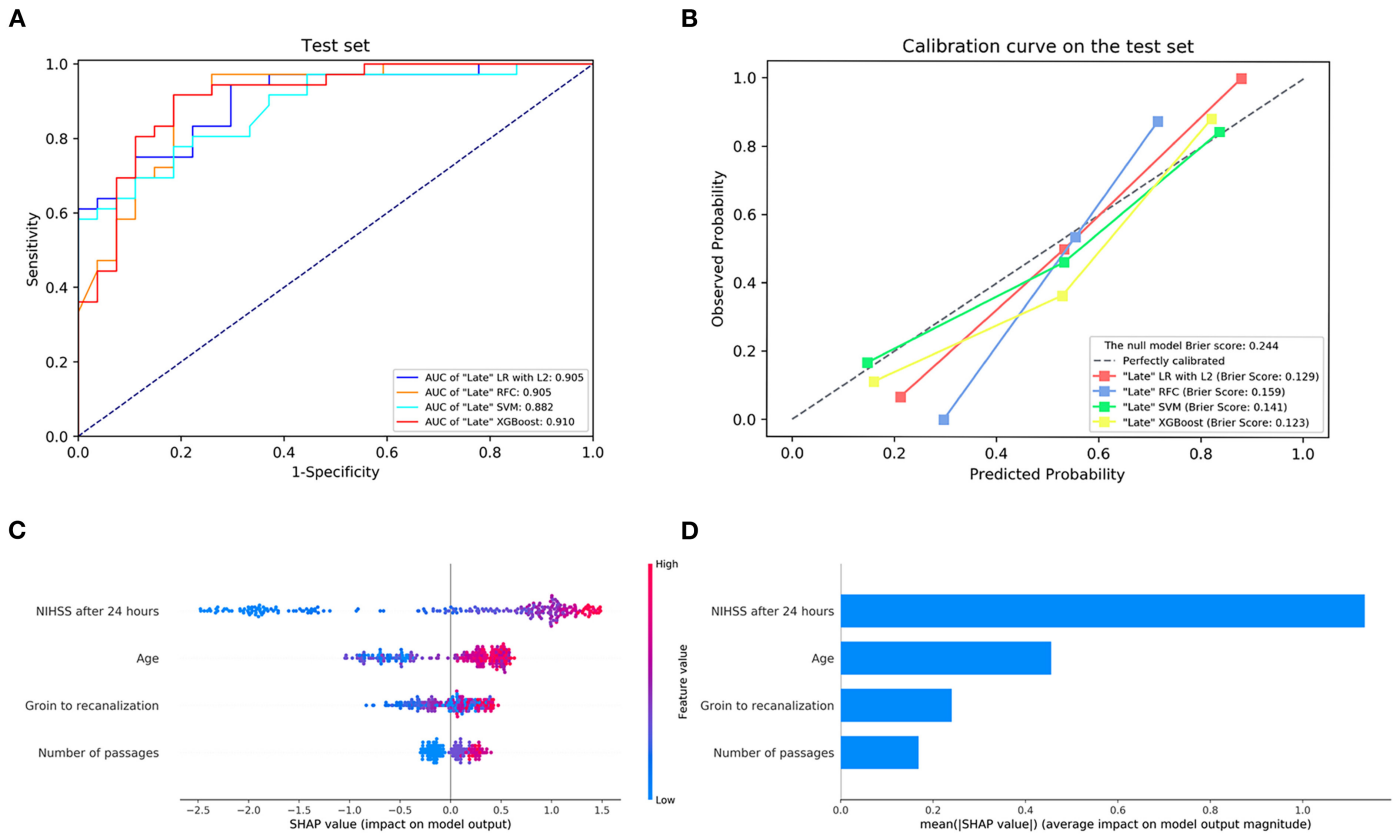
**(A)** The receiver operating characteristic curve and **(B)** the calibration curve of the “Late” machine learning models on the testing set. **(C,D)** Feature importance ranking based on Shapley Additive exPlanations (SHAP) values in “Late” XGBoost. AUC, area under the curve; LR with L2, logistic regression with L2 regularization; SVM, support vector machine; RFC, random forest classifier; XGBoost, extreme gradient boost. NIHSS, National Institutes of Health Stroke Scale.

The details of the model's performance on the training set are provided in Supplementary Figure S4 and Supplementary Table S3B. In the testing step, all “Late” model also scored a similar AUC since the statistical insignificant differences of AUCs were found {AUC of 0.910 [95% confidence intervals (CI), 0.837–0.984] for XGBoost vs. 0.905 [95% CI, 0.834–0.976] for LR, 0.905 [95% CI, 0.829–0.981] for RFC and 0.882 [95% CI, 0.801–0.962] for SVM}. And homoplastically, the overall performance of the XGBoost model outperformed other ML models with the consideration of the smaller Brier score (0.123 for XGBoost vs. 0.129 for LR, 0.159 for RFC, and 0.141 for SVM). A summary of the results is given in Figure 2, Table 2B, and Supplementary Table S4B.

**Table 2B:** Scores of each “Late” model on the test set.

**Model**	**AUC (95% CI)**	**Sensitivity**	**Specificity**	**PPV**	**NPV**	**Accuracy**	**Brier score**
LR with L2	0.905 (0.834–0.976)	0.889	0.704	0.800	0.826	0.810	0.129
RFC	0.905 (0.829–0.981)	0.917	0.815	0.868	0.880	0.873	0.159
SVM	0.882 (0.801–0.962)	0.889	0.630	0.762	0.810	0.778	0.141
XGBoost	0.910 (0.837–0.984)	0.861	0.815	0.861	0.815	0.841	0.123

AUC, the area under the receiver operating characteristic curve; CI, confidence intervals; PPV: positive predictive value; NPV, negative predictive value; LR with L2, logistic regression with L2 regularization; RFC, random forest classifier; SVM, support vector machine; XGBoost, extreme gradient boosting.

**Figure 3:**
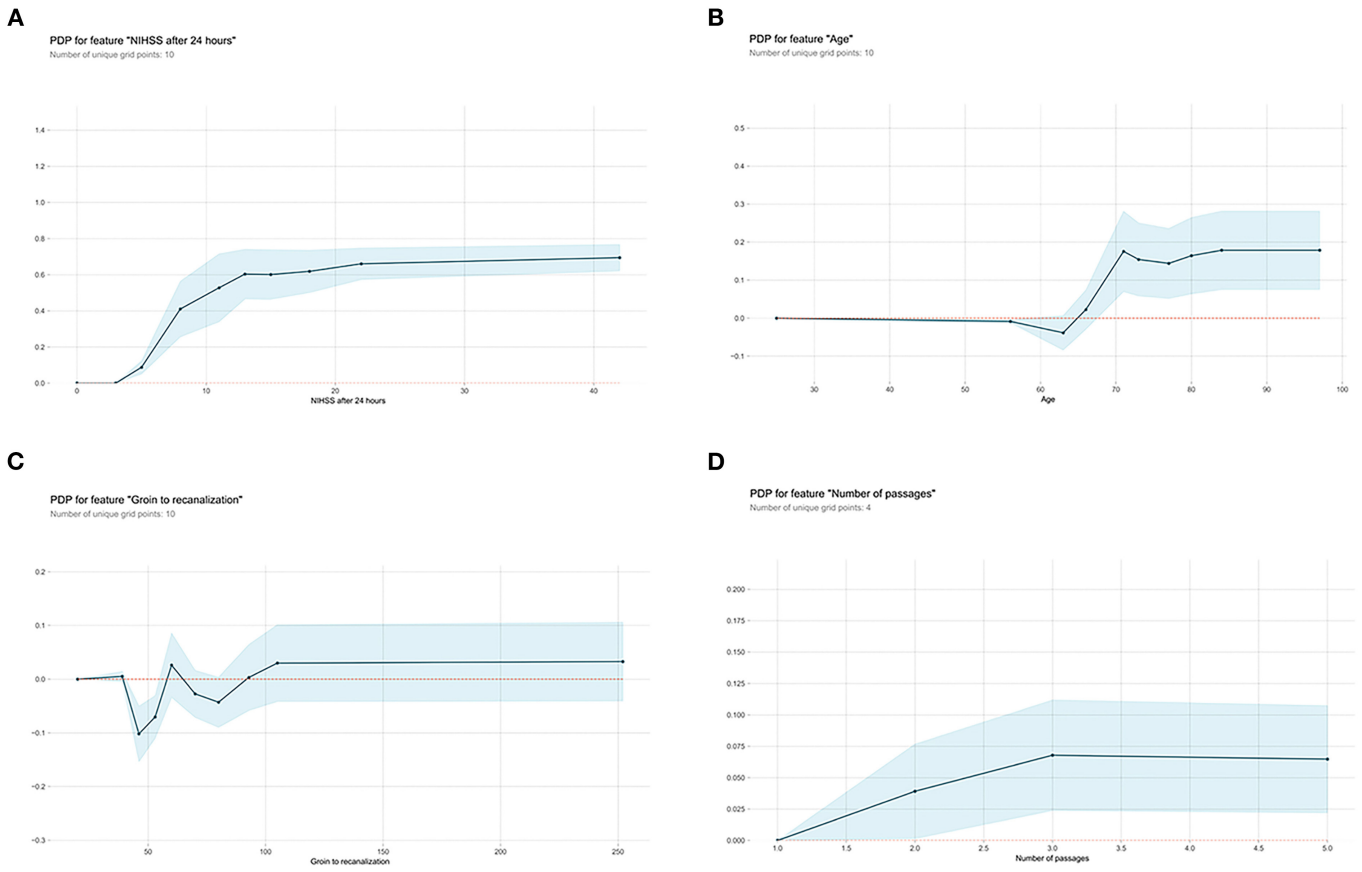
Partial dependence plots (PDP) of “Late” XGBoost model features. **(A)** NIHSS after 24 hours, **(B)** age, **(C)** groin to recanalization, and **(D)** the number of passages. The shaded blue region shows the magnitude of the confidence interval, and the Y-axis represents the change in the predicted outcome. NIHSS, National Institutes of Health Stroke Scale.

Next, the visual interpretation of the optimal “Late” model (XGBoost) was provided. Figure 2C shows that NIHSS after 24 h, age, groin to recanalization, and the number of passages were the four important features. And the high SHAP values of these four features revealed positive contributions to futile recanalization. Furthermore, from the PDP, we can see that for NIHSS after 24 h (Figure 3A), the increase in NIHSS score (3–22) is positively related to futile recanalization and remains stable when NIHSS is over 22. For the age feature (Figure 3B), on average, the increase in age (63–71) is positively related to futile recanalization, which performs similarly to the “Early” model but with less fluctuation. For the groin to recanalization feature (Figure 3C), on average, it (46–105) was positively related to futile recanalization, and then the impact remains constant after groin to recanalization of 105 min. For the number of passages feature (Figure 3D), the impact increased gradually with the number from one to three and remained stable when the number was over three.

### Comparison of models

**Figure 4:**
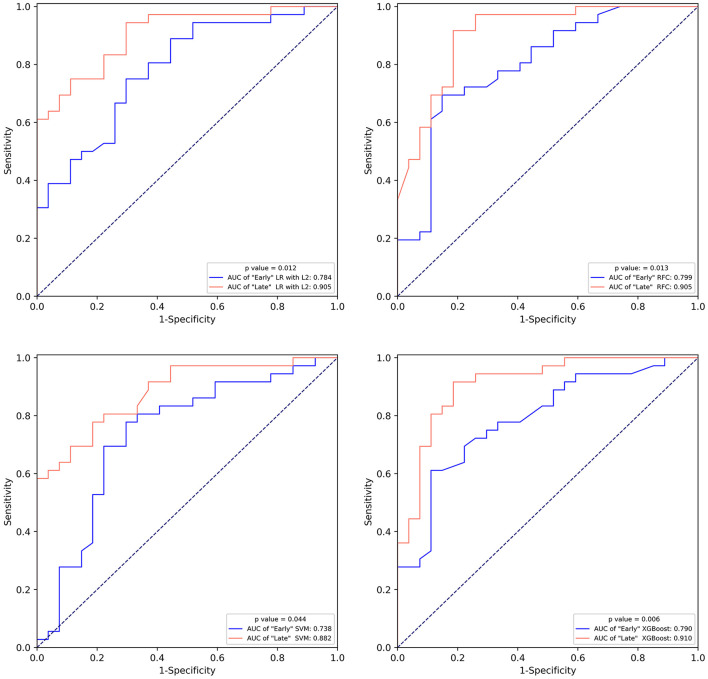
The comparison of the receiver operating characteristic curve of “Early” machine learning models and “Late” machine learning models on the test set. LR with L2, logistic regression with L2 regularization; SVM, support vector machine; RFC, random forest classifier; XGBoost, extreme gradient boost.

We compared the performance of the “Early” and “Late” models (Figure 4; Supplementary Figure S5). All “Late” models substantially outperformed “Early” models on both the training and testing set.

## Discussion

In the present study, we derived and validated a series of ML models with the capacity to predict futile recanalization in LVO patients undergoing EVT. The XGBoost algorithm has optimal predictive performance in both the “Early” model and the “Late” model. As a result, we generated highly reliable futile recanalization risk estimates and made predictions at two points (pre- and post-EVT) in the care continuum with the explicit goal of improving stroke emergency care. In addition, results in our study suggest that the inclusion of peri-interventional characteristics may lead to superior predictive performance compared to a model based on baseline characteristics only. Although several pieces of literature have reported many prognostic factors associated with futile recanalization, none integrated those factors for building predictive models. Considering the hazards of futile recanalization, such models are important.

Using “Early” XGBoost may offer neurologists effective support in patient selection for EVT therapy. According to the HERMES meta-analysis, “the number needed to treat with endovascular thrombectomy to reduce disability by at least one level on Mrs for one patient was 2.6” if the clinical trial criteria were used (2). In real-world practice, however, a higher number would be needed given the potential benefit for a portion of patients. Hence, patient selection criteria for EVT tend to be more liberal, often accompanied by disastrous futile recanalization. Reliable pre-EVT prognostic tools can facilitate the process of patient selection by generating an accurate prediction of futile recanalization. However, it must be admitted that although the “Early” XGBoost constructed in our study achieved an AUC of 0.790, this model needs further improvement due to the existence of the “smoking paradox.” On the other hand, the “Late” XGBoost with the inclusion of peri-interventional characteristics outperformed the “Early” XGBoost by a margin of 12.0% for AUC. The accurate prediction provided reliable and objective prognostic after EVT and, in turn, can aid in the counseling of patients and their relatives.

There are two points to emphasize, with the expectation that the “Early” XGBoost and the “Late” XGBoost can be integrated into real-world practice. On the one hand, because of the irreplaceability of clinical judgment, the proposed use for the “Early” XGBoost and the “Late” XGBoost is to serve as adjuncts, rather than surrogates, to clinical judgment to facilitate evidence-based, prediction-driven, and personalized decision-making in the clinical workflow of LVO patients. On the other hand, this study represents only one component in the development of robust and reliable tools for the risk screening of futile recanalization in LVO patients. Further implementation, impact, and validation studies are essential if those models are going to be integrated into the clinical workflow.

In addition to clinical applications, results in our study can provide meaningful insights to reveal diverse and new predictors. The SHAP algorithm and PDP have discovered several predictors of futile recanalization. SHAP algorithm can provide feature importance scores from XGBoost, and explain the logic behind predictions; PDP was used to show the marginal effect of a single feature. When XGBoost integrated information from baseline clinical and radiological characteristics before EVT, the SHAP algorithm demonstrated that—in the present study—patients presenting with greater age and with severe neurological deficit on admission had higher rates of futile recanalization. Nevertheless, this of course does not suggest that EVT is not indicated in patients with a higher baseline NIHSS. It is essential to consider the results of a meta-analysis of five randomized trials, which provides evidence of no differential benefit from endovascular treatment across the entire NIHSS severity range (2). Although it might be surprising at first glance, current smoking was associated with decreased risk of futile recanalization in the present study. The so-called “smoking paradox” phenomenon has appeared in patients undergoing intravenous thrombolysis and EVT (17, 18). One assumption was that the negative relation between current smoking and futile recanalization was related to an age effect (19). Indeed, current smokers were younger than former smokers or never smokers in the present study (*p* < 0.001) (Supplementary Table S5). However, such a result must not be misinterpreted that the effect of smoking is beneficial due to the observational study design. Surprisingly, the ML algorithm did not select ASPECTS on admission as one of the predictors for futile recanalization. Such a result can be explained by the firm, linear, negative correlation between ASPECTS and NIHSS on admission and the higher univariate performance of the latter as compared with the former for predicting futile recanalization (20). Unfortunately, pre-treatment collateral status was not incorporated in the model building process due to its unavailability in the dataset. Previous studies have shown the positive effect of good collateral status on clinical outcomes after EVT (21, 22). Such results can be explained by the fact that more robust collateral flow can compensate for the brain areas with restricted blood flow and subsequently increase the recanalization and reperfusion rates (23). However, these studies did not consider the degree of revascularization achieved. According to a study published in the Stroke journal (23), reperfusion success is associated with good collateral status, which indicates that the effect of collateral status on clinical outcome is possibly indirect. In addition, patients in the present study who received EVT and recanalization achieved an mTICI score of >2a, which means that those patients are likely to have good collateral status.

Subsequent inclusion of peri-interventional characteristics into the XGBoost demonstrated that NIHSS after 24 h, age, groin to recanalization, and the number of passages were the key predictors for futile recanalization. As shown in the PDP, in the age group between 63 and 71, there was an abrupt rise of futile recanalization by around 20% independent of other patient characteristics, while it remains stable at other ages. Although age is known to be prominent for the efficacy of EVT (3, 4), the observed drastic change in the probability of futile recanalization has not been described before. Interestingly, the “late” XGBoost with peri-interventional characteristics shows that NIHSS assessed at 24 h replaced NIHSS on admission and became the strongest predictor of futile recanalization. Indeed, as demonstrated by previous studies, NIHSS after 24 h is strongly associated with long-term functional outcomes and is a great potential early surrogate clinical endpoint for clinical trials (10, 24). For the groin to recanalization feature (Figure 2C), on average, the increase in the groin to recanalization (46–105 min) is positively related to futile recanalization. Such results are in line with those from previous studies which flagged a time dependency to clinic outcome of LVO stroke treated with EVT (25, 26). Also, the likelihood of futile recanalization got sequentially higher as the number of passages increased. Although the technical expertise of the operator is important, the increased number of passages may be due to some uncontrollable factors such as increased clot fragmentation with distal embolization or accumulated endothelial damage (27). In addition, the impact of the groin to recanalization and the number of passages on futile recanalization supports the conclusion of previous studies that an angiographic recanalization does not necessarily lead to functional independence if it was achieved at the expense of longer procedural times (28).

The present study has some limitations. Firstly, the patients included in the present study were selected from a single-center, retrospective data set, leading to selection bias. For example, older age patients are more likely to be excluded from EVT. Indeed, such selection bias is inherent in any prediction model. Secondly, the definition of successful reperfusion itself should be challenged as it is assessed on the final angiogram. Because our study design was retrospective, we could not reliably confirm which patients went on to develop spontaneous re-occlusion or recanalization at 24–48 h. However, this phenomenon is only present in a small percentage of patients. Thirdly, other significant data sources, such as angiographic characteristics, are likely to add extra predictive value. We did not include these in our study because such data were unavailable and may be a logistical challenge when applied, especially in the primary stroke centers.

## Conclusions

The “Early” XGBoost and the “Late” XGBoost allowed us to accurately predict futile recanalization in LVO patients before and after EVT. Our study suggests that the inclusion of peri-interventional characteristics may lead to superior predictive performance compared to a model based on baseline characteristics only. In addition, NIHSS after 24 h was the most important prognostic factor for futile recanalization. Although our results represent only one step in developing screening tools for futile recanalization, they can provide meaningful insights to reveal diverse and new prognostic factors.

## Data availability statement

The original contributions presented in the study are included in the article/Supplementary material, further inquiries can be directed to the corresponding authors.

## Ethics statement

The scientific use of the data was approved by the Ethics Committee of Nanjing First Hospital (document number: KY20130424-01) and all patients provided written informed consent.

## Author contributions

XL formed the conception and study design. XC, QJ, YW, and RQ did the data collection. ZZ and XP did the data analysis. JZho and NC did the literature review. XZ and DZ did the model development. XL, XZ, and JZha drafted the manuscript. LT, CG, and JZo made significant revisions and supplied valuable improvement suggestions. All authors approved the final version. All authors have read and agreed to the published version of the manuscript.

## Funding

This study was supported by the National Natural Science Foundation of China (82173899), and Jiangsu Pharmaceutical Association (H202108 and A2021024).

## Conflict of interest

The authors declare that the research was conducted in the absence of any commercial or financial relationships that could be construed as a potential conflict of interest.

## Publisher's note

All claims expressed in this article are solely those of the authors and do not necessarily represent those of their affiliated organizations, or those of the publisher, the editors and the reviewers. Any product that may be evaluated in this article, or claim that may be made by its manufacturer, is not guaranteed or endorsed by the publisher.

## References

[1] Powers WJ Rabinstein AA Ackerson T Adeoye OM Bambakidis NC Becker K. Guidelines for the Early Management of Patients With Acute Ischemic Stroke: 2019 Update to the 2018 Guidelines for the Early Management of Acute Ischemic Stroke: A Guideline for Healthcare Professionals From the American Heart Association/American Stroke Association. Stroke. (2019) 50:e344–418. 10.1161/STR.0000000000000211 31662037

[2] Goyal M Menon BK van Zwam WH Dippel DW Mitchell PJ Demchuk AM. Endovascular thrombectomy after large-vessel ischaemic stroke: a meta-analysis of individual patient data from five randomised trials. Lancet. (2016) 387:1723–31. 10.1016/S0140-6736(16)00163-X 26898852

[3] van Horn N Kniep H Leischner H McDonough R Deb-Chatterji M Broocks G. Predictors of poor clinical outcome despite complete reperfusion in acute ischemic stroke patients. J Neurointerv Surg. (2021) 13:14–8. 10.1136/neurintsurg-2020-015889 32414889

[4] Zhou T Yi T Li T Zhu L Li Y Li Z. Predictors of futile recanalization in patients undergoing endovascular treatment in the DIRECT-MT trial. J Neurointervent Surg. (2021) 14:752–5. 10.1136/neurintsurg-2021-017765 34475255

[5] Pan H Lin C Chen L Qiao Y Huang P Liu B. Multiple-factor analyses of futile recanalization in acute ischemic stroke patients treated with mechanical thrombectomy. Front Neurol. (2021) 12:704088. 10.3389/fneur.2021.704088 34489851 PMC8416752

[6] Hussein HM Georgiadis AL Vazquez G Miley JT Memon MZ Mohammad YM. Occurrence and predictors of futile recanalization following endovascular treatment among patients with acute ischemic stroke: a multicenter study. Am J Neuroradiol. (2010) 31:454–8. 10.3174/ajnr.A2006 20075087 PMC7963981

[7] Nie X Pu Y Zhang Z Liu X Duan W Liu L. Futile Recanalization after endovascular therapy in acute ischemic stroke. Biomed Res Int. (2018) 2018:5879548. 10.1155/2018/5879548 29854767 PMC5966674

[8] Hussein HM Saleem MA Qureshi AI. Rates and predictors of futile recanalization in patients undergoing endovascular treatment in a multicenter clinical trial. Neuroradiology. (2018) 60:557–63. 10.1007/s00234-018-2016-2 29574600

[9] Xu H Jia B Huo X Mo D Ma N Gao F. Predictors of futile recanalization after endovascular treatment in patients with acute ischemic stroke in a multicenter registry study. J Stroke Cerebrovasc Dis. (2020) 29:105067. 10.1016/j.jstrokecerebrovasdis.2020.105067 32912569

[10] Brugnara G Neuberger U Mahmutoglu MA Foltyn M Herweh C Nagel S. Multimodal predictive modeling of endovascular treatment outcome for acute ischemic stroke using machine-learning. Stroke. (2020) 51:3541–51. 10.1161/STROKEAHA.120.030287 33040701

[11] Kamel H Navi BB Parikh NS Merkler AE Okin PM Devereux RB. Machine learning prediction of stroke mechanism in embolic strokes of undetermined source. Stroke. (2020) 51:e203–10. 10.1161/STROKEAHA.120.029305 32781943 PMC8034802

[12] Qiu W Kuang H Teleg E Ospel JM Sohn SI Almekhlafi M. Machine learning for detecting early infarction in acute stroke with non-contrast-enhanced CT. Radiology. (2020) 294:638–44. 10.1148/radiol.2020191193 31990267

[13] DeLong ER DeLong DM Clarke-Pearson DL. Comparing the areas under two or more correlated receiver operating characteristic curves: a nonparametric approach. Biometrics. (1988) 44:837–45. 10.2307/2531595 3203132

[14] Karhade AV Schwab JH Bedair HS. Development of machine learning algorithms for prediction of sustained postoperative opioid prescriptions after total hip arthroplasty. J Arthroplasty. (2019) 34:2272–7.e2271. 10.1016/j.arth.2019.06.013 31327647

[15] Friedman JH. Greedy function approximation: a gradient boosting machine. Ann Stat. (2001) 29:1189–232. 10.1214/aos/1013203451

[16] Singh R Lanchantin J Sekhon A Qi Y. Attend and Predict: Understanding gene regulation by selective attention on chromatin. Adv Neural Inf Process Syst. (2017) 30:6785–95. 10.1101/329334 30147283 PMC6105294

[17] von Martial R Gralla J Mordasini P El Koussy M Bellwald S Volbers B. Impact of smoking on stroke outcome after endovascular treatment. PLoS ONE. (2018) 13:e0194652. 10.1371/journal.pone.0194652 29718909 PMC5931491

[18] Kvistad CE Oeygarden H Logallo N Thomassen L Waje-Andreassen U Naess H. Is smoking associated with favourable outcome in tPA-treated stroke patients? Acta Neurol Scand. (2014) 130:299–304. 10.1111/ane.12225 24527872

[19] Hussein HM Niemann N Parker ED Qureshi AI. Searching for the Smoker's paradox in acute stroke patients treated with intravenous thrombolysis. Nicotine Tob Res. (2017) 19:871–6. 10.1093/ntr/ntx020 28339617

[20] Kent DM Hill MD Ruthazer R Coutts SB Demchuk AM Dzialowski I. “Clinical-CT mismatch” and the response to systemic thrombolytic therapy in acute ischemic stroke. Stroke. (2005) 36:1695–9. 10.1161/01.STR.0000173397.31469.4b 16002756

[21] Román LS Menon BK Blasco J Hernández-Pérez M Dávalos A Majoie C. Imaging features and safety and efficacy of endovascular stroke treatment: a meta-analysis of individual patient-level data. Lancet Neurology. (2018) 17:895–904. 10.1016/S1474-4422(18)30242-4 30264728

[22] Leng X Fang H Leung TW Mao C Miao Z Liu L. Impact of collaterals on the efficacy and safety of endovascular treatment in acute ischaemic stroke: a systematic review and meta-analysis. J Neurol Neurosurg Psychiatry. (2016) 87:537–44. 10.1136/jnnp-2015-310965 26063928

[23] Liebeskind DS Jahan R Nogueira RG Zaidat OO Saver JL. Impact of collaterals on successful revascularization in Solitaire FR with the intention for thrombectomy. Stroke. (2014) 45:2036–40. 10.1161/STROKEAHA.114.004781 24876081 PMC4157911

[24] Mistry EA Yeatts S de Havenon A Mehta T Arora N De Los Rios La Rosa F. Predicting 90-day outcome after thrombectomy: baseline-adjusted 24-hour NIHSS is more powerful than NIHSS score change. Stroke. (2021) 52:2547–53. 10.1161/STROKEAHA.120.032487 34000830 PMC11261999

[25] Saver JL Goyal M van der Lugt A Menon BK Majoie CB Dippel DW. Time to treatment with endovascular thrombectomy and outcomes from ischemic stroke: a meta-analysis. JAMA. (2016) 316:1279–88. 10.1001/jama.2016.13647 27673305

[26] Jahan R Saver JL Schwamm LH Fonarow GC Liang L Matsouaka RA. Association between time to treatment with endovascular reperfusion therapy and outcomes in patients with acute ischemic stroke treated in clinical practice. JAMA. (2019) 322:252–63. 10.1001/jama.2019.8286 31310296 PMC6635908

[27] Arai D Ishii A Chihara H Ikeda H Miyamoto S. Histological examination of vascular damage caused by stent retriever thrombectomy devices. J Neurointerv Surg. (2016) 8:992–5. 10.1136/neurintsurg-2015-011968 26508129

[28] Kitano T Todo K Yoshimura S Uchida K Yamagami H Sakai N. Futile complete recanalization: patients characteristics and its time course. Sci Rep. (2020) 10:4973. 10.1038/s41598-020-61748-y 32188911 PMC7080727

