# Corrigendum: Machine learning to predict futile recanalization of large vessel occlusion before and after endovascular thrombectomy

**DOI:** 10.3389/fneur.2024.1499029

**Published:** 2024-10-11

**Authors:** Xinping Lin, Xiaohan Zheng, Juan Zhang, Xiaoli Cui, Daizu Zou, Zheng Zhao, Xiding Pan, Qiong Jie, Yuezhang Wu, Runze Qiu, Junshan Zhou, Nihong Chen, Li Tang, Chun Ge, Jianjun Zou

**Affiliations:** ^1^School of Basic Medicine and Clinical Pharmacy, China Pharmaceutical University, Nanjing, China; ^2^Department of Pharmacy Nanjing First Hospital, Nanjing Medical University, Nanjing, China; ^3^Department of Neurology, Nanjing Yuhua Hospital, Yuhua Branch of Nanjing First Hospital, Nanjing Medical University, Nanjing, China; ^4^Department of Pharmacy, Nanjing First Hospital, China Pharmaceutical University, Nanjing, China; ^5^Department of Neurology, Nanjing First Hospital, Nanjing Medical University, Nanjing, China; ^6^Department of Pharmacy, Yixing Cancer Hospital, Yixing, China

**Keywords:** futile recanalization, machine learning, large vessel occlusion, endovascular thrombectomy, predictive model

In the published article, there was an error in Supplementary Table 1. The authors accidentally uploaded the incorrect Supplementary Table 1 that contains non-public information of patients.

The updated Supplementary Table has been published.

The authors apologize for this error and state that this does not change the scientific conclusions of the article in any way. The original article has been updated.

