# Supplementary material for: Machine learning to predict futile recanalization of large vessel occlusion before and after endovascular thrombectomy
Source: Front Neurol. 2022 Aug 19;13:909403. doi: 10.3389/fneur.2022.909403 (PMC9437637; doi:10.3389/fneur.2022.909403)
Supplement: Supplementary file 2 [file Data_Sheet_1.docx]

**Supplementary Methods**

**Data collection and definitions**

Stroke causes were categorized into five different subtypes in accordance with Trial of Org 10172 in Acute Stroke Treatment (TOAST) [1]. Dyslipidemia was diagnosed if two or more of the following symptoms occurred: (1) total cholesterol ≥ 200mg /dL (5.20 mmol/L); (2) triglyceride ≥ 150mg/dL (1.70 mmol/L); (3) low-density lipoprotein ≥ 120mg /dL (3.12 mmol/L); (4) high-density lipoprotein ≤ 35mg /dL (0.91 mmol/L) [2]. Early neurological deterioration was defined as an increase of 4 points or more in postprocedural NIHSS score within 24 h from baseline. Per the European Cooperative Acute Stroke Study (ECASS) II definition, symptomatic intracranial hemorrhage referred to any postprocedural hemorrhage with an increment in NIHSS score of over 4 points [3].

BP collection

Data on BP were collected at several time points after EVT treatment: at 1, 3, 6, 12, and 24 hours post-EVT treatment.

SD was defined as the dispersion of a dataset relative to its mean and is calculated as the square root of the variance by determining the variation between each data point relative to the mean according to following equation:

$$SD=\sqrt{\frac{1}{n-1} \sum_{i=1}^{n-1} \left( BPi-{BP}_{mean} \right)^{2}}$$

CV was defined as the ratio of the SD and the mean according to following equation:

$$CV=\frac{SD}{mean}*100[\%]$$

[1] Adams HP Jr, Bendixen BH, Kappelle LJ, et al. Classification of subtype of acute ischemic stroke. Definitions for use in a multicenter clinical trial. TOAST. Trial of Org 10172 in Acute Stroke Treatment. Stroke. 1993;24(1):35-41. https://doi.org/10.1161/01.str.24.1.35.

[2] Vekic J, Zeljkovic A, Stefanovic A, Jelic-Ivanovic Z, Spasojevic-Kalimanovska V. Obesity and dyslipidemia. Metabolism. 2019; 92:71-81. https://doi.org/10.1016/j.metabol.2018.11.005.

[3] Hacke W, Kaste M, Fieschi C, et al. Randomised double-blind placebo-controlled trial of thrombolytic therapy with intravenous alteplase in acute ischaemic stroke (ECASS II). Second European-Australasian Acute Stroke Study Investigators. Lancet. 1998;352(9136):1245-1251. doi:10.1016/s0140-6736(98)08020-9.

**Supplementary Tables**

**Table S1-A** Hyperparameters of ML-based “Early” models.

| **model** | **Hyper-parameters** | **value** |
| --- | --- | --- |
| LR with L2 |  |  |
|  | C | 0.029 |
|  | Class weight | 0:1; 1:1 |
| RFC |  |  |
|  | N estimators | 140 |
|  | Max depth | 3 |
|  | Min samples leaf | 15 |
|  | Min samples split | 70 |
|  | Class weight | 0:1; 1:1 |
| SVM |  |  |
|  | C | 0.3 |
|  | Gamma | 1 |
|  | Kernel | rbf |
|  | Class weight | 0:1; 1:1 |
| XGBoost |  |  |
|  | Max depth | 3 |
|  | Learning rate | 0.04 |
|  | N estimators | 60 |
|  | Min child weight | 8 |
|  | Gamma | 0.5 |
|  | Subsample | 0.9 |
|  | Colsample bytree | 0.5 |
|  | Scale pos weight | 1 |

Abbreviations: ML, machine learning; LR with L2, logistic regression with L2 regularization; RFC, random forest classifer; SVM, support vector machine; XGBoost, extreme gradient boosting. All options were left as default other than Hyper-parameters in table.

**Table S1-B** Hyperparameters of ML-based “Late” models.

| **model** | **Hyper-parameters** | **value** |
| --- | --- | --- |
| LR with L2 |  |  |
|  | C | 0.081 |
|  | Class weight | 0:1; 1:1 |
| RFC |  |  |
|  | N estimators | 120 |
|  | Max depth | 3 |
|  | Min samples leaf | 5 |
|  | Min samples split | 140 |
|  | Class weight | 0:1; 1:1 |
| SVM |  |  |
|  | C | 0.1 |
|  | Gamma | 0.082 |
|  | Kernel | rbf |
|  | Class weight | 0:1; 1:1 |
| XGBoost |  |  |
|  | Max depth | 5 |
|  | Learning rate | 0.04 |
|  | N estimators | 70 |
|  | Min child weight | 2 |
|  | Gamma | 0.4 |
|  | Subsample | 0.68 |
|  | Colsample bytree | 0.76 |
|  | Scale pos weight | 1 |

Abbreviations: ML, machine learning; LR with L2, logistic regression with L2 regularization; RFC, random forest classifer; SVM, support vector machine; XGBoost, extreme gradient boosting. All options were left as default other than Hyper-parameters in table.

**Table S2-A** Demographics and clinical characteristics of the training and test set.

|  | **Training set**  **(n = 249, 80%)** | **Test set**  **(n = 63, 20%)** | **p-value** |
| --- | --- | --- | --- |
| Futile recanalization | 143(57.4) | 36(57.1) | 0.967 |
| Baseline characteristics |  |  |  |
| Age, years, median (IQR) | 72(64-79) | 69(62-80) | 0.933 |
| Male sex, n (%) | 148(59.4) | 38(60.3) | 0.899 |
| BMI, kg/m^2^, median (IQR) | 23.88(21.37-26.3) | 24.22(22.04-27.06) | 0.184 |
| Education, years, n (%) |  |  | 0.918 |
| 0–6 | 141(56.6) | 33(52.4) |  |
| 6–9 | 53(21.3) | 14(22.2) |  |
| 9–12 | 33(13.3) | 9(14.3) |  |
| >12 | 22(8.8) | 7(11.1) |  |
| Premorbid mRS (IQR) | 0(0-0) | 0(0-0) | 0.571 |
| NIHSS on admission, median (IQR) | 14(11-18) | 13(10-17) | 0.349 |
| Baseline SBP, mmHg, mean (SD) | 138.84(22.97) | 134.81(24.22) | 0.220 |
| Baseline DBP, mmHg, mean (SD) | 84.26(14.72) | 83.05(16.16) | 0.567 |
| Risk factors of vessels |  |  |  |
| Hypertension, n (%) | 191(76.7) | 45(71.4) | 0.383 |
| Diabetes mellitus, n (%) | 77(30.9) | 24(38.1) | 0.277 |
| Dyslipidemia, n (%) | 61(24.5) | 15(23.8) | 0.909 |
| Coronary artery disease, n (%) | 52(20.9) | 10(15.9) | 0.373 |
| Atrial fibrillation, n (%) | 78(31.3) | 21(33.3) | 0.760 |
| Previous ischemic stroke/TIA, n (%) | 52(20.9) | 15(23.8) | 0.613 |
| Previous hemorrhagic stroke, n (%) | 3(1.2) | 1(1.6) | 1.000 |
| Smoking, n (%) |  |  | 0.810 |
| Never smoker | 154(61.8) | 37(58.7) |  |
| Former smoker | 19(7.6) | 4(6.3) |  |
| Current smoker | 76(30.5) | 22(34.9) |  |
| Drinking, n (%) |  |  | 0.969 |
| Never drinker | 178(71.5) | 46(73) |  |
| Former drinker | 12(4.8) | 3(4.8) |  |
| Current drinker | 59(23.7) | 14(22.2) |  |
| Radiological baseline characteristics |  |  |  |
| ASPECTS on admission, median (IQR) | 5(4-6) | 5(4-7) | 0.690 |
| Cause of stroke, n (%) |  |  |  |
| LAA | 99(39.8) | 22(34.9) | 0.481 |
| CE | 122(49) | 36(57.1) | 0.248 |
| SAO | 4(1.6) | 0(0) | 0.586 |
| SOC | 7(2.8) | 1(1.6) | 1.000 |
| SUC | 17(6.8) | 4(6.3) | 1.000 |
| Vascular occlusion site, n (%) |  |  |  |
| ICA | 80(32.1) | 17(27) | 0.431 |
| MCA M1 | 150(60.2) | 44(69.8) | 0.160 |
| MCA M2 | 19(7.6) | 2(3.2) | 0.269 |
| Side of occlusion, n (%) |  |  |  |
| Left | 120(48.2) | 27(42.9) | 0.448 |
| Right | 117(47) | 34(54) | 0.322 |
| Both side | 12(4.8) | 2(3.2) | 0.743 |
| Medication use history |  |  |  |
| Previous antiplatelet, n (%) | 36(14.5) | 7(11.1) | 0.491 |
| Previous anticoagulation, n (%) | 19(7.6) | 7(11.1) | 0.372 |
| Previous statin, n (%) | 24(9.6) | 5(7.9) | 0.678 |

Abbreviations: IQR, interquartile range; SD, standard deviation; BMI, body mass index; mRS, modified Ranking Scale; NIHSS, National Institutes of Health Stroke Scale; SBP, systolic blood pressure; DBP, diastolic blood pressure; TIA, transient ischemic attacks; ASPECTS, Alberta Stroke Program Early CT Score; LAA, large artery atherosclerosis; CE, cardioembolism; SAO, small artery occlusion; SOC, stroke of other determined cause; SUC, stroke of undetermined cause; ICA, internal carotid artery; MCA, middle cerebral artery.

**Table S2-B** Treatment information and Complication of the training and test set.

|  | **Training set**  **(n = 249, 80%)** | **Test set**  **(n = 63, 20%)** | **p-value** |
| --- | --- | --- | --- |
| Treatment information |  |  |  |
| Intravenous thrombolysis, n (%) | 114(45.8) | 24(38.1) | 0.272 |
| Number of passages, n (%) | 2(1-3) | 1(1-3) | 0.989 |
| Onset to emergency, min, median (IQR) | 150(60-295) | 135(70-235) | 0.637 |
| Onset to image, min, median (IQR) | 190(116-335) | 213(140-309) | 0.933 |
| Onset to groin, min, median (IQR) | 260(185-410) | 257(200-360) | 0.843 |
| Onset to recanalization, min, median (IQR) | 344(245-490) | 340(270-430) | 0.916 |
| Groin to recanalization, min, median (IQR) | 64(49-89) | 65(45-84) | 0.952 |
| Later than 6h from onset to puncture, n (%) | 78(31.3) | 15(23.8) | 0.244 |
| Later than 8h from onset to puncture, n (%) | 48(19.3) | 7(11.1) | 0.129 |
| mTICI score, n (%) |  |  | 0.462 |
| 2b | 98(39.4) | 28(44.4) |  |
| 3 | 151(60.6) | 35(55.6) |  |
| NIHSS after 24 hours, median (IQR) | 12(5-17) | 12(7-18) | 0.606 |
| Post-treatment blood pressure variability |  |  |  |
| SBP |  |  |  |
| SD, median (IQR) | 11.55(7.98-16.71) | 11.52(6.19-17.50) | 0.275 |
| CV, median (IQR) | 0.09(0.06-0.13) | 0.08(0.05-0.13) | 0.217 |
| DBP |  |  |  |
| SD, median (IQR) | 8.40(5.80-11.19) | 8.66(4.67-11.22) | 0.503 |
| CV, median (IQR) | 0.11(0.08-0.15) | 0.11(0.07-0.15) | 0.309 |
| Complications |  |  |  |
| Brain edema, n (%) | 12(4.8) | 2(3.2) | 0.573 |
| END_24_, n (%) | 28(11.2) | 11(17.5) | 0.183 |
| sICH, n (%) | 7(2.8) | 2(3.2) | 1.000 |

Abbreviations: IQR, interquartile range; mTICI, modified Thrombolysis in Cerebral Infarction; SBP, systolic blood pressure; DBP, diastolic blood pressure; SD, standard deviation; CV, coefficient of variation; NIHSS, National Institutes of Health Stroke Scale; END, early neurological deterioration; sICH, symptomatic intracranial hemorrhage.

**Table S3-A** Scores of each “Early” model on the training set.

| **Model** | **AUC (95% CI)** | **Sensitivity** | **Specificity** | **PPV** | **NPV** | **Accuracy** | **Brier score** |
| --- | --- | --- | --- | --- | --- | --- | --- |
| LR with L2 | 0.747(0.686-0.809) | 0.811 | 0.585 | 0.725 | 0.697 | 0.715 | 0.205 |
| RFC | 0.781(0.723-0.839) | 0.825 | 0.632 | 0.752 | 0.728 | 0.743 | 0.195 |
| SVM | 0.781(0.721-0.840) | 0.797 | 0.679 | 0.770 | 0.713 | 0.747 | 0.184 |
| XGBoost | 0.792(0.736-0.847) | 0.657 | 0.830 | 0.839 | 0.642 | 0.731 | 0.191 |

Abbreviations: AUC, the area under the receiver operating characteristic curve; CI, confidence intervals; PPV: positive predictive value; NPV, negative predictive value; LR with L2, logistic regression with L2 regularization; RFC, random forest classiﬁer; SVM, support vector machine; XGBoost, extreme gradient boosting.

**Table S3-B** Scores of each “Late” model on the training set.

| **Model** | **AUC (95% CI)** | **Sensitivity** | **Specificity** | **PPV** | **NPV** | **Accuracy** | **Brier score** |
| --- | --- | --- | --- | --- | --- | --- | --- |
| LR with L2 | 0.871(0.827-0.914) | 0.825 | 0.764 | 0.825 | 0.764 | 0.799 | 0.150 |
| RFC | 0.892(0.852-0.931) | 0.888 | 0.745 | 0.825 | 0.832 | 0.827 | 0.162 |
| SVM | 0.865(0.821-0.910) | 0.867 | 0.717 | 0.805 | 0.800 | 0.803 | 0.148 |
| XGBoost | 0.945(0.919-0.971) | 0.895 | 0.868 | 0.901 | 0.860 | 0.884 | 0.101 |

Abbreviations: AUC, the area under the receiver operating characteristic curve; CI, confidence intervals; PPV: positive predictive value; NPV, negative predictive value; LR with L2, logistic regression with L2 regularization; RFC, random forest classiﬁer; SVM, support vector machine; XGBoost, extreme gradient boosting.

**Table S4-A** P-value for comparing AUC of different “Early” models on the test set.

| **Model** | **LR** | **RFC** | **SVM** | **XGBoost** |
| --- | --- | --- | --- | --- |
| LR with L2 | \ | 0.626 | 0.257 | 0.841 |
| RFC | 0.626 | \ | 0.057 | 0.570 |
| SVM | 0.257 | 0.057 | \ | 0.187 |
| XGBoost | 0.841 | 0.570 | 0.187 | \ |

Abbreviations: AUC, the area under the receiver operating characteristic curve; LR with L2, logistic regression with L2 regularization; RFC, random forest classiﬁer; SVM, support vector machine; XGBoost, extreme gradient boosting.

**Table S4-B** P-value for comparing AUC of different “Late” models on the test set.

| **Model** | **LR** | **RFC** | **SVM** | **XGBoost** |
| --- | --- | --- | --- | --- |
| LR with L2 | \ | 1.000 | 0.122 | 0.847 |
| RFC | 1.000 | \ | 0.507 | 0.745 |
| SVM | 0.122 | 0.507 | \ | 0.396 |
| XGBoost | 0.847 | 0.745 | 0.396 | \ |

Abbreviations: AUC, the area under the receiver operating characteristic curve; LR with L2, logistic regression with L2 regularization; RFC, random forest classiﬁer; SVM, support vector machine; XGBoost, extreme gradient boosting.

**Table S5** Comparison of the age with different smoking status

|  | **Total n = 312** | **Never smoker n = 191** | **Former smoker n = 23** | **Current smoker n = 98** | **p-value** |
| --- | --- | --- | --- | --- | --- |
| Age | 72.00(63.25-79.00) | 75.00(67.00-82.00) | 73.00(67.00-78.00) | 65.00(56.75-73.00) | < 0.001 |

**Supplementary Figures**


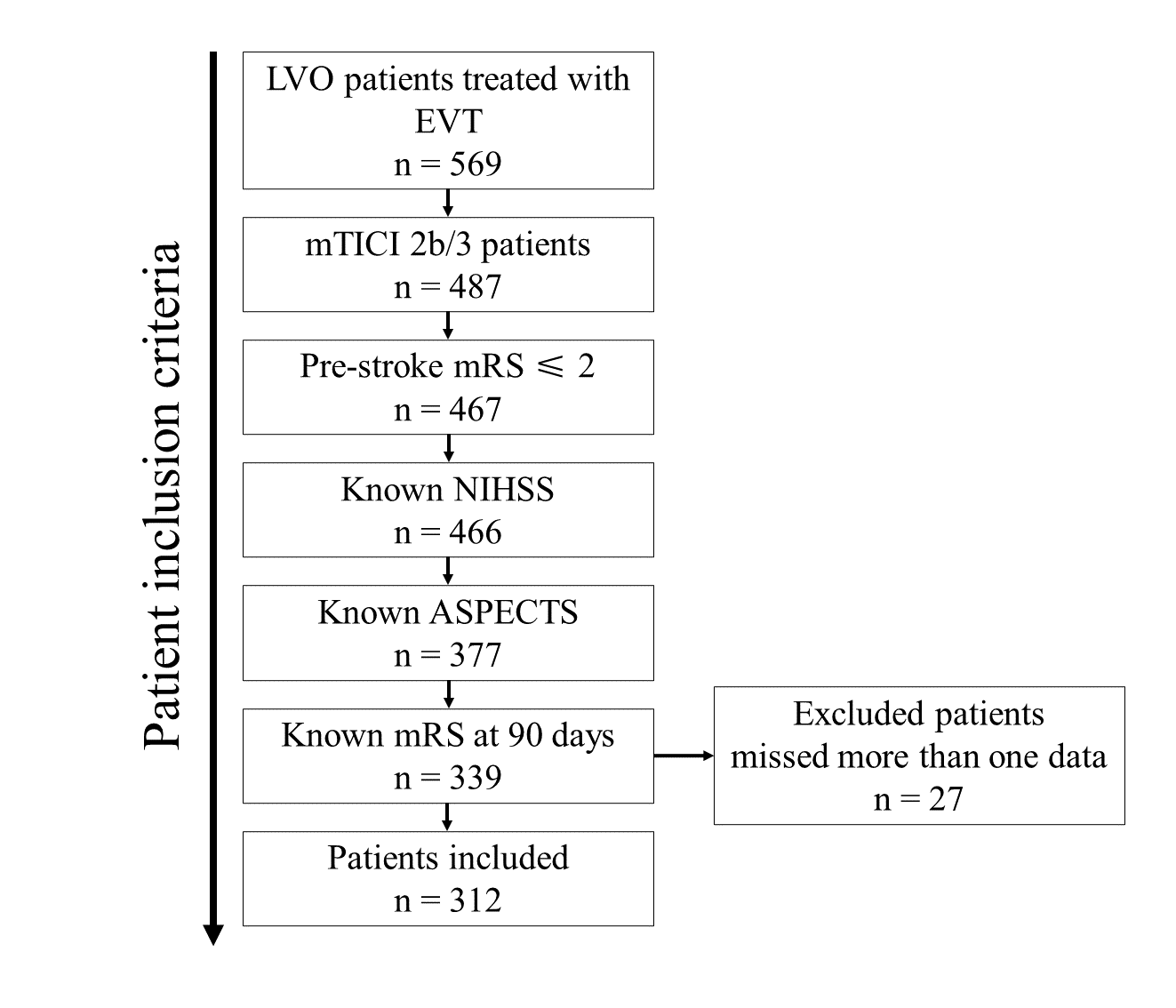


**Figure S1**. The patient flowchart. LVO, large vessel occlusion; EVT, endovascular thrombectomy; mTICI, modified Thrombolysis In Cerebral Infarction; mRS, modified Rankin Scale; NIHSS, National Institutes of Health Stroke Scale; ASPECTS, Alberta Stroke Program Early Computed Tomography Score

**
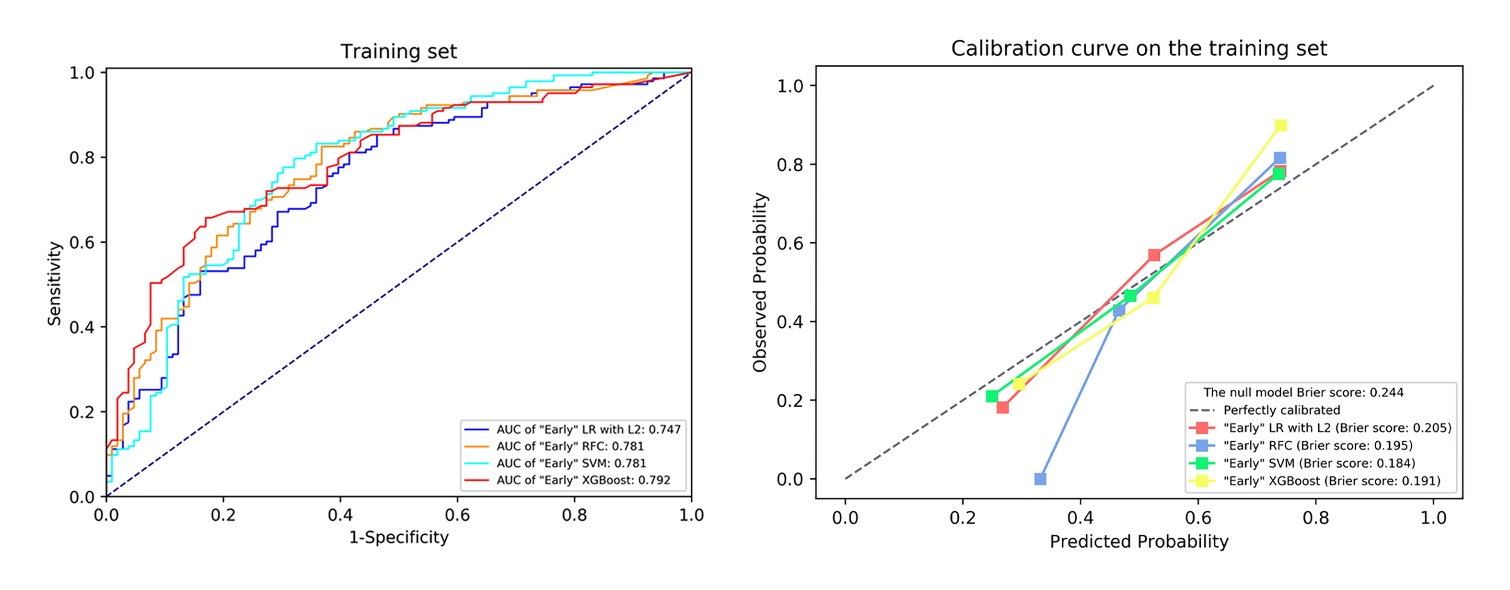
**

**Figure S2.** Receiver operating characteristic curves (ROC) and the calibration curve of “Early” models on the training set. Abbreviations: AUC, area under the curve; LR with L2, logistic regression with L2 regularization; RFC, random forest classifier; SVM, support vector machine; XGBoost, extreme gradient boosting.


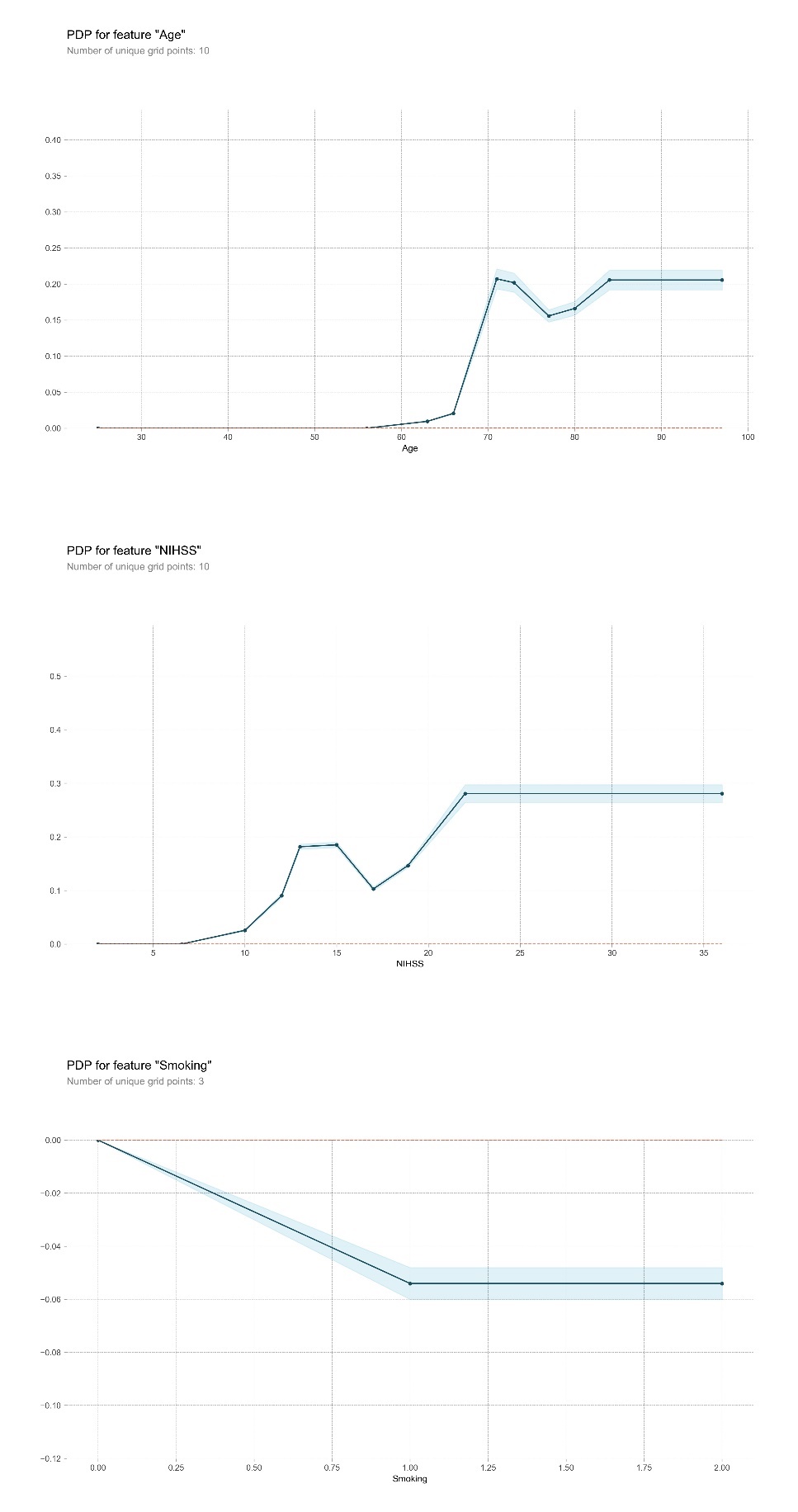


**Figure S3.** Partial dependence plots (PDP) of “Final” XGBoost model features. NIHSS, National Institutes of Health Stroke Scale

**Figure S4.** Receiver operating characteristic curves (ROC) and the calibration curve of “Late” models on the training set. Abbreviations: AUC, area under the curve; LR with L2, logistic regression with L2 regularization; RFC, random forest classifier; SVM, support vector machine; XGBoost, extreme gradient boosting.


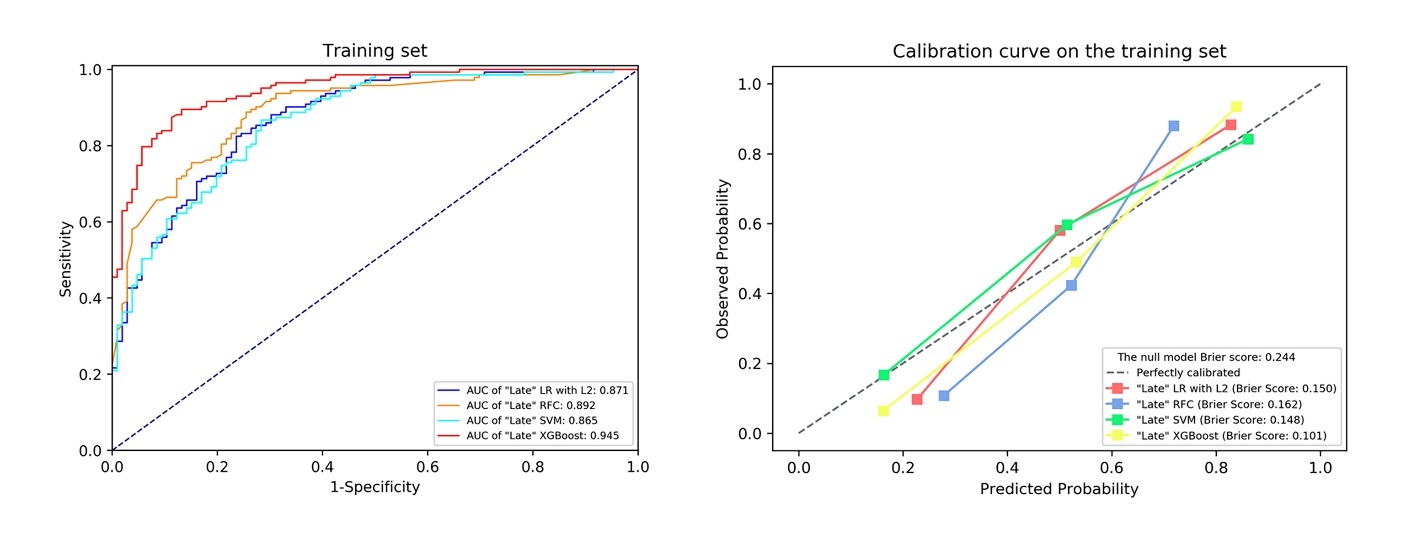


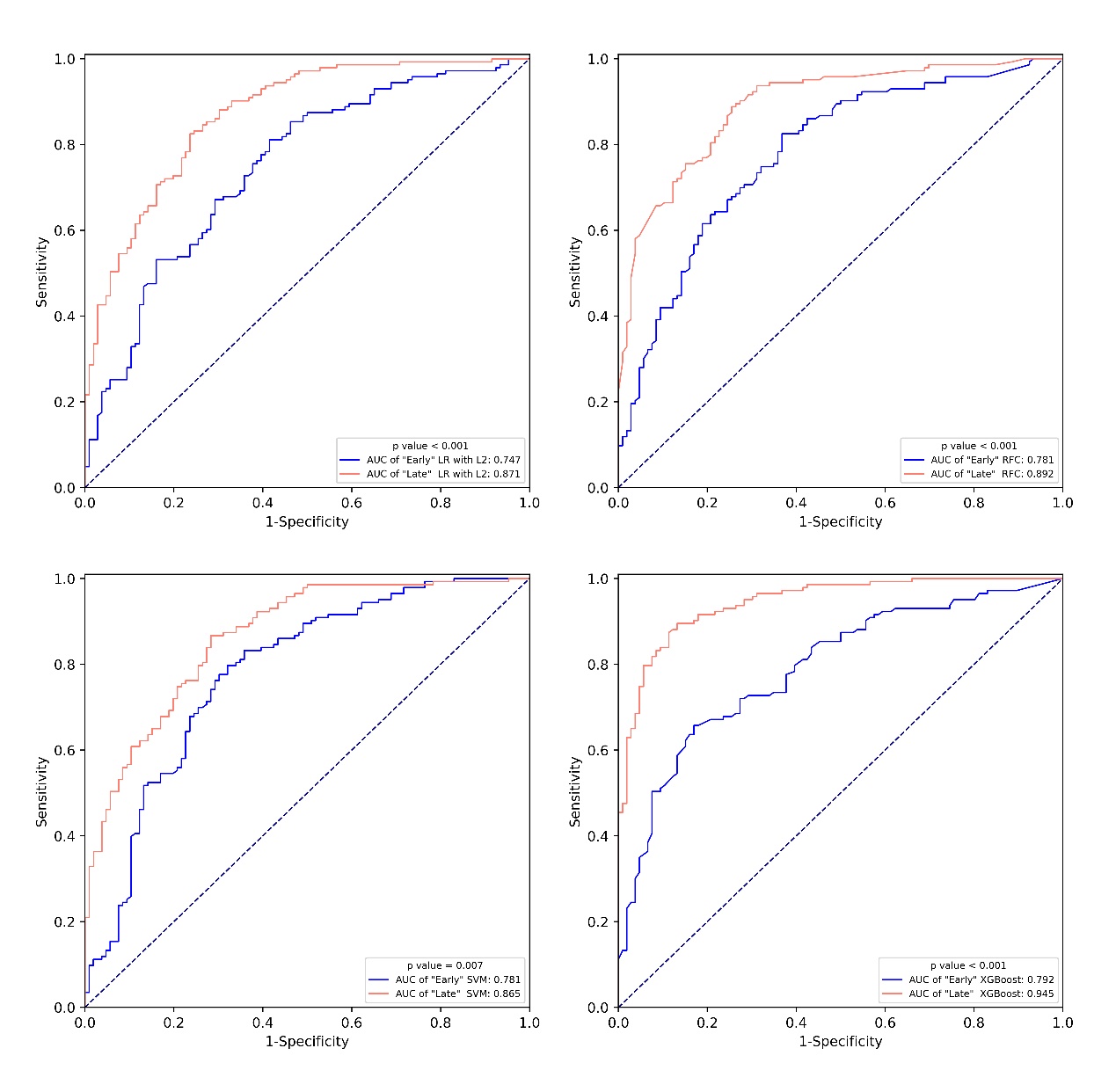


**Feature S5.** The comparison of the receiver operating characteristic curve (ROC) of “Early” machine learning models and “Late” machine learning models on the training set. AUC, area under the curve; LR with L2, logistic regression with L2 regularization; RFC, random forest classifier; SVM, support vector machine; XGBoost, extreme gradient boosting.
